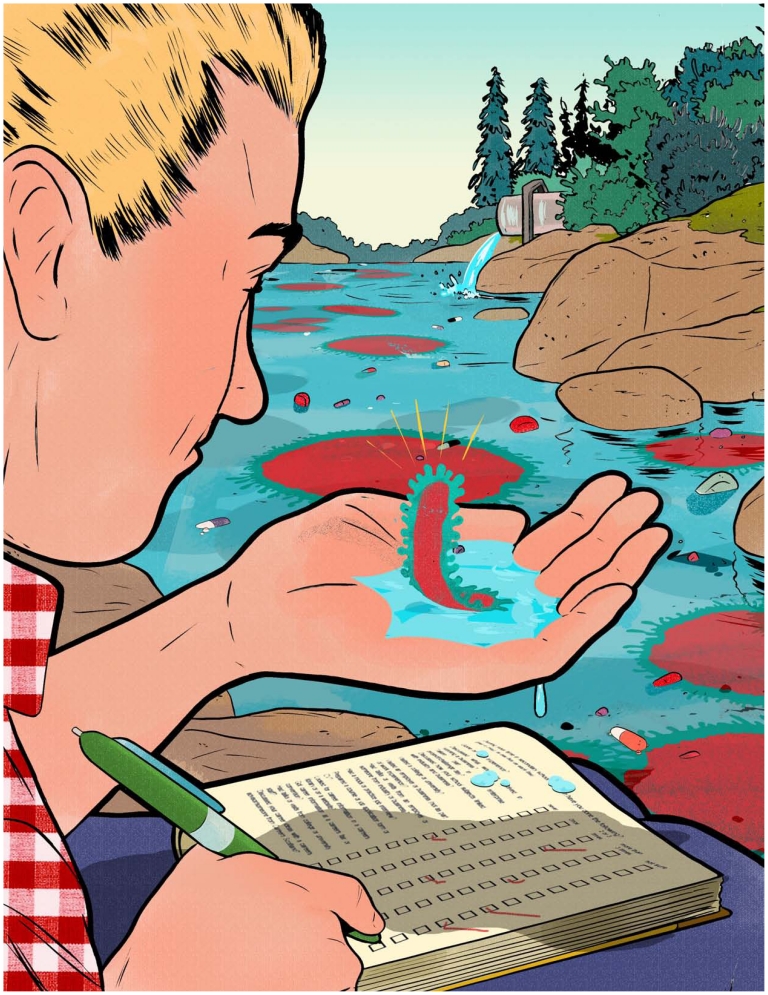# Tools for Tracking Antibiotic Resistance

**DOI:** 10.1289/ehp.119-a214

**Published:** 2011-05

**Authors:** Naomi Lubick

**Affiliations:** **Naomi Lubick** is a freelance science writer based in Stockholm, Sweden, and Folsom, CA. She has written for *Environmental Science & Technology*, *Nature*, and *Earth*

When a team of researchers from Sweden first started measuring chemicals in a river near Patancheru, India, they found shocking concentrations of drugs flowing downstream—for example, levels of the potent antibiotic ciprofloxacin greater than those found in the blood of humans taking the drug. A major source of these drugs was treated wastewater from pharmaceutical manufacturing plants that was discharged into the river and surrounding environs, as Joakim Larsson and his colleagues from the University of Gothenburg reported several years ago.^1^ An update published in *PLoS ONE*^2^ now links the drugs with downstream development of microbes with genetic resistance to multiple antibiotics typically used to treat human illness.

The researchers found snippets of genetic material in bacteria from river sediments downstream of the treatment plant that conferred resistance not only to ciprofloxacin, a fluoroquinolone, but also to betalactams, aminoglycosides, sulfonamides, and other classes of antibiotics. Several genes that provide resistance to ciprofloxacin and have the ability to transfer between different bacteria were extremely common at some of the sampling sites.^2^

What if the bacteria in Patancheru could develop ways to survive the daily onslaught of ciprofloxacin, most likely over the course of years in their river environment, and ended up passing on their new genetic resistance to pathogenic bacteria that could be a threat to human health? Although Larsson’s team has yet to catalog antibiotic resistance in the local population, people in the region are continually exposed to resistant microbes as they use the river water for agriculture and everyday home life. “This is a huge scary experiment in nature,” Larsson says.

Just how isolated these kinds of drug “hot spots” are remains unknown, although researchers have pressed for global monitoring of antibiotic use and resistance for the past several decades, across disciplines as diverse as clinical medicine and ecotoxicity. Bringing together these fields reflects the breadth of challenges in tracking antibiotic resistance, but new technologies and ideas hold promise for the near future.

## Overcoming a Lack of Coordination

“Misuse of antibiotics is obviously what creates the basic factors that produce drug resistance,” says Mario Raviglione, director of the World Health Organization (WHO) department charged with tuberculosis control; this is true in both the developing and developed worlds. And despite educational campaigns by the U.S. Centers for Disease Control and Prevention (CDC)^3^ and others aimed at improving clinicians’ use of antibiotics, overprescribing remains a problem for multiple reasons.^4^ Moreover, patient compliance—for example, taking the full course of prescribed antibiotics—can be lax, which leads to the evolution of more antibiotic-resistant pathogens.

Agricultural use of human drugs adds to the threat of drug resistance. After World War II, antibiotics started to be used for purposes such as growth promotion in livestock. Since then, antibiotics—and in some cases, the genes for resistance to multiple drugs—have been found on industrial cattle, swine, and shrimp farms,^5^,^6^,^7^,^8^ measured on chicken skins in grocery stores,^9^ and even detected in apple orchards sprayed with drugs originally intended for human use.^10^

For World Health Day in April 2011 the WHO chose the theme of the global spread of antibiotic resistance, marking a little over a decade since the organization first called for patient and doctor guidelines to protect antibiotics from becoming obsolete.^11^ A document issued by the WHO in 2001 put forth a series of recommendations for patients and the general community, prescribers and dispensers, hospitals, agricultural enterprises, national governments and health systems, and drug developers and promoters.^12^ However, in general “very few countries, if any, have made a comprehensive effort to do any of the measures included in the older guidelines,” says Raviglione, who led preparations for World Health Day 2011. “Why are countries not picking them up? Lack of resources? Their health systems are not strong enough? The cost of drugs?”

On 7 April 2011 the organization released updated policy guidance for countries to curb the spread of antibiotic resistance in health care settings.^13^ Some of this guidance is aimed at doctors and hospitals, while some is geared toward policy makers and legislators. The simple package of policy recommendations is intended to be easy for countries to adopt, Raviglione says. The WHO-level focus on antibiotic resistance issues also gives health ministers around the world a platform from which to call for funding and research attention at home.

But individual countries cannot handle this issue acting alone. During its 2009 presidency of the European Union, the Swedish government highlighted antibiotic resistance, focusing the conversation on solutions across Europe.^14^ For instance, a September 2009 meeting targeted industry, policy makers, and others focused on finding incentives for creating new drugs.^15^ Meanwhile, in the United States, the U.S. Food and Drug Administration (FDA) in 2010 issued draft guidance urging the judicious use of medically important antibiotics in livestock.^16^ The 2011–2015 Strategic Plan of the National Antimicrobial Resistance Monitoring System, a collaboration between the FDA, CDC, U.S. Department of Agriculture, and state and local health departments, includes efforts to strengthen sampling, reporting, and international and domestic collaborative efforts.^17^

Bills aimed at addressing antibiotic resistance were introduced in 2009 in the House of Representatives^18^ and the Senate,^19^ but foundered. This year, Louise Slaughter (D–NY) tried again in the House, introducing H.R. 965, the Preservation of Antibiotics for Medical Treatment Act of 2011^20^ on March 9. Another bill in the House, H.R. 6331, the Generating Antibiotic Incentives Now Act of 2010,^21^ would create incentives to bring new antibiotics to market by speeding up the approval process. These bills have lingered in committee, even as the problem continues to grow globally.

## Traveling Wild

The antibiotic drug hot spot in India is not alone: researchers have traced drugs flowing downstream from manufacturers in China and Cuba^22^ and from wastewater treatment plants in the United States.^23^,^24^ They also have identified antimicrobial resistance genetic material in treated waste effluent and tap water in Michigan and Ohio,^25^,^26^ and researchers in Sweden recently documented multidrug-resistant *Escherichia coli* in the waste of migrating birds in the Arctic.^27^

Given the widespread presence of antibiotics in the natural environment,^28^ it should not be a surprise that resistance is growing in wild bacteria and microbes—but is that resistance transferable to pathogens relevant to human health? Eventually the answer will most certainly be yes: Bacteria, microbes, and even fungi under stress from high concentrations of drugs might have the ability to replicate DNA snippets and possibly pass them on to other microbial species in the environment, says Dave Ussery, an associate professor of microbial genomics at the Technical University of Denmark. Transported in the integrons, plasmids, and other cellular genetic modules that confer resistance, these snippets can be traded like baseball cards among microbes.^29^

The locations and impacts of reservoirs of antibiotic resistance in the wild remain enigmatic,^30^ and not only for humans. Antibiotic drugs and any acquired resistance to them might, for example, affect how microbes communicate with each other through “quorum sensing,” a protein signaling feedback loop key to microbial population dynamics.^31^

But until recently, truly wild settings have garnered less interest from researchers, policy makers, and media than agricultural exposures—for example, farmers who handle pigs treated with multiple drugs and then end up with resistant strains on their skin,^32^ including methicillin-resistant *Staphylococcus aureus*.

Researchers from the U.S. Geological Survey led by Dana Kolpin also are looking at animal waste for how much residual concentration of antibiotic it might carry and the potential impacts on microbial life if the manure is spread on agricultural fields or released accidentally. Results of Kolpin’s research are not yet available, but a small-scale study by another group tracking resistance genes from biosolids and manure at two soil sites has shown the genes—with resistance for tetracycline and sulfanomides—transfer at different rates depending on soil type.^33^

## Kink in the Pipeline

Another worry underlying the issue of resistance is the fact that pharmaceutical companies are not discovering new antibiotics. At least three reasons explain why the pipeline is so empty, says Ingrid Petersson, director of science relations at pharmaceutical company AstraZeneca. First, finding new pathways in microbes or pinpointing proteins to create new antibiotics is difficult, in part because of what could be considered an embarrassment of possibilities with too many unknowns. Second, she says, the regulatory environment is complicated: getting a drug through approval processes takes a long time and costs a lot of money, among other factors. And third, low prices for existing antibiotics—many of which are generics—do not encourage companies to invest in new drugs.

“Existing, older antibiotics are cheaper, which makes it difficult to achieve realistic prices for new antibiotics—prices which would provide a viable return on investment,” explains Colin Mackay, director of communication and partnerships for the European Federation of the Pharmaceutical Industries and Associations, a trade organization. “Furthermore, antibiotics are only used acutely, perhaps only for a week to ten days at a time. This adds to the difficulty in making a return on investment. By comparison, treatments for chronic illnesses, say for cardiovascular or musculoskeletal conditions, are used long term, perhaps for the rest of the patient’s life.”

“Drug development . . . is one of the most critical things that we are facing,” says Otto Cars, the chairman of ReAct (Action on Antibiotic Resistance), an independent think tank, and a professor of infectious diseases at Uppsala University. For gut flora that can shift readily from animal to human hosts—including Gram-negative^34^ enterics such as *Salmonella*, *Campylobacter*, *Klebsiella*, *E. coli*, and *Shigella*—horizontal gene transfer is “moving rapidly now,” he says. “These kinds of infections that Gram-negative bacteria are causing are already untreatable; even in the rich part of the world, there are totally resistant strains,” he says. “The drug pipeline is particularly empty for that space.”

Nevertheless, some major companies are looking into new antibiotics. For example, AstraZeneca is looking for solutions for multidrug-resistant tuberculosis.^35^,^36^ Companies are investing money, sometimes by acquiring smaller companies that have begun the research or joining in efforts with nonprofits^37^ and academic researchers. Petersson notes that the Trans Atlantic Task Force on Antimicrobial Resistance,^38^ formed between the European Union and the United States as part of the 2009 EU–US Summit Declaration,^39^ will present suggestions at this year’s summit meeting for areas of cooperation, including incentives for industry to pursue new drug development.

The WHO, the CDC, and other national, international, and nonprofit organizations are pursuing alternative business models. Government funding, as when a federal agency invests in vaccine research, may be one option, Cars suggests. So-called advanced market commitments—where governments pledge to purchase drugs, thus guaranteeing a market—is another option. In its new policy guidance,^13^ the WHO calls for global and national commitments to develop drugs and share information on the national costs of inaction, as well as “push” and “pull” incentives to reduce the inherent risks in the initial phases of research and development and to offset the risks of an uncertain market, respectively.

## Crossing Boundaries

Heeding calls^40^,^41^,^42^ for better management of the drugs currently available to doctors will require much more attention to trends of resistance. In particular, monitoring is now lacking. “If anything, we don’t know enough about developing countries to understand the situation—what resistant bacteria are there? In Europe and the U.S., systems of surveillance are in place, but not in most of Africa or Asia,” Raviglione says, referring to health systems, although the same holds true for environmental surveys.

Europe and the United States use far more antibiotics by volume as well as newer antibiotics compared with less affluent countries that typically use fewer and older generations of drugs, Raviglione says. That would indicate such countries might not yet have resistance to latest-generation drugs the same way Europe and the United States do. But there remains concern about the outsourcing of drug production to developing countries, especially India and China, where lax enforcement of regulations could increase the likelihood of unchecked environmental releases of active pharmaceutical ingredients—hence studies such as Larsson’s work in Patancheru. The potential for impacts of manufacturing newer antibiotics in developing countries, with possible unwanted environmental releases, has not yet been studied, say the scientists contacted for this story.

Making data internationally available so that teams are tracking the same genes and species in different countries may be one avenue of attack on the issue of antibiotic resistance. Julian Davies, a professor emeritus of microbiology and immunology at the University of British Columbia, and David Graham, an environmental engineer at Newcastle University, have been working on a proposal to bring together members of the medical, environmental, and microbial research communities to address antibiotic resistance issues. If funded, their efforts could result in local centers on every continent working to monitor antibiotic resistance, look for simple solutions to preventing the spread of antibiotic-resistant microbes inside hospitals, and communicate about antibiotic resistance issues at an international scale.

The interdisciplinary breadth needed to address the many issues at hand have led to miscommunications stemming from vocabulary, Graham says. Take the word *transmission*, he explains: “To a physician or engineer, transmission means migration between individuals at larger scales, whereas to a microbiologist transmission is something at the micro-scale between the bacteria themselves. It took us [the diverse members of the team working on the proposal] awhile to agree upon a common language.”

But microbes have few such communication barriers, and they quickly find ways to communicate resistance, as plane transit brings countries—and antibiotic resistance hot spots—closer together. Some of the global aspects of the problem can be illustrated by last year’s description of NDM-1, a protein present on plasmids that confers antibiotic resistance to multiple antibiotics.^43^ The mechanism of resistance traveled from hospitals in India to the United Kingdom via patients who had visited the subcontinent, presumably for cheap medical treatments, and then returned home. Furthermore, NDM-1 has appeared in tap water and wastewater outside of hospital settings in New Delhi, according to another recent report in *The Lancet Infectious Diseases*,^44^ heightening concerns for local transmission in an urban environment.

## Tools for the Trade

Ussery and his colleagues, while working to figure out antibiotic resistance transfer at a basic level, are also developing field tools for tracking antibiotic resistance genes and the microbes that carry them. The team includes computer scientists searching for simple algorithms that will let researchers make fast identifications of resistance gene sequences and resistant microbial species.

Ussery envisions a device on every doctor’s desk that could take a swab from a patient and sequence the DNA from that sample. This device, connected to the Internet or with databases preloaded, could use the sequence to identify the microbes present to the genus or even species level, then spot genes they might carry for resistance to certain drugs. The test could potentially even predict the effectiveness of specific drugs in individual patients. That could guide doctors in prescribing treatment or hospitals in determining when to quarantine a patient with a particularly virulent resistant strain of an infectious disease.

“Some machines are now doing single-molecule sequencing [of bacteria] from one cell. The technology is almost there,” Ussery says, citing manufacturer Oxford Nanopore’s machines that can sequence a microbe from one cell as being close to market-ready.

For tracking antibiotic resistance in the field, these techniques could prove to be a massive boon, but they will have to become much less expensive than current market costs of the machines, comments Davies. The newest so-called third-generation apparatuses for identifying genetic codes in the blink of an eye may cut the cost of reading an entire genome to a tenth or less of current costs, but machines will still cost hundreds of thousands of dollars, he says, backed up by the price tag of Pacific Biosciences’ first entry to the market late last year.

## Searching for Simple Answers

Even a very expensive diagnostic tool would save a lot of lives, Cars says, and it would also save antibiotics for clinical use: rapid tests used on a daily basis could lead to hospital practices that save time and money while conserving antibiotics for treatment.

But new technologies are not the only tool that will be necessary to address all the threads that intertwine the problem of spreading antibiotic resistance, Cars and others say. Tracking genes in hospitals and the wild, putting policies in place to limit use of antibiotics outside of absolute necessity in a clinical setting—the wish list goes on.

And yet, in some ways, simple solutions seem to be in the offing. U.S. congressmen recently visited Denmark to learn more about that country’s successful transition away from antibiotic use for growth promotion in swine. Contrary to stories about how Denmark’s agricultural sector crashed after that use of antibiotics completely stopped five years ago, Frank Møller Aarestrup of the Technical University of Denmark and colleagues report the country today continues to increase its exports of pigs without the help of antibiotics, depending solely on improved animal husbandry techniques.^45^

This anecdote underscores the power of seemingly simple policy decisions to make immediate changes with promising consequences. Absent such courageous steps, “it’s almost just a matter of time” before antibiotic resistance is transferred to a pathogen that matters for human health, Ussery says.

“It’s an enormously frustrating situation,” says Davies, who says he feels he and his colleagues talk a lot about the problem without making much headway at the larger scale. In the end, he says, solutions can only come from convincing the general public and lawmakers that the time to act is now.

## Figures and Tables

**Figure:**